# Breast cancer recurrence after reoperation for surgical bleeding

**DOI:** 10.1002/bjs.10592

**Published:** 2017-08-07

**Authors:** R N Pedersen, K Bhaskaran, U Heide-Jørgensen, M Nørgaard, P M Christiansen, N Kroman, H T Sørensen, D P Cronin-Fenton

**Affiliations:** Department of Clinical Epidemiology, Aarhus University, Aarhus, Denmark; Breast and Endocrine Section, Department of Surgery P, Aarhus University Hospital, Aarhus, Denmark; Danish Breast Cancer Group, Copenhagen, Denmark; Department of Breast Surgery, Rigshospitalet, Copenhagen, Denmark; Department of Non-Communicable Disease Epidemiology, London School of Hygiene and Tropical Medicine, London, UK

## Abstract

**Background:**

Bleeding activates platelets that can bind tumour cells, potentially promoting metastatic growth in patients with cancer. This study investigated whether reoperation for postoperative bleeding is associated with breast cancer recurrence.

**Methods:**

Using the Danish Breast Cancer Group database and the Danish National Patient Register (DNPR), a cohort of women with incident stage I–III breast cancer, who underwent breast-conserving surgery or mastectomy during 1996–2008 was identified. Information on reoperation for bleeding within 14 days of the primary surgery was retrieved from the DNPR. Follow-up began 14 days after primary surgery and continued until breast cancer recurrence, death, emigration, 10 years of follow-up, or 1 January 2013. Incidence rates of breast cancer recurrence were calculated and Cox regression models were used to quantify the association between reoperation and recurrence, adjusting for potential confounders. Crude and adjusted hazard ratios according to site of recurrence were calculated.

**Results:**

Among 30 711 patients (205 926 person-years of follow-up), 767 patients had at least one reoperation within 14 days of primary surgery, and 4769 patients developed breast cancer recurrence. Median follow-up was 7·0 years. The incidence of recurrence was 24·0 (95 per cent c.i. 20·2 to 28·6) per 1000 person-years for reoperated patients and 23·1 (22·5 to 23·8) per 1000 person-years for non-reoperated patients. The overall adjusted hazard ratio was 1·06 (95 per cent c.i. 0·89 to 1·26). The estimates did not vary by site of breast cancer recurrence.

**Conclusion:**

In this large cohort study, there was no evidence of an association between reoperation for bleeding and breast cancer recurrence.

## Teaser

No correlation

## Introduction

Breast cancer is the most common cancer among women, with about 1·67 million new patients diagnosed in 2012^1^. With 522 000 annual breast cancer-related deaths estimated worldwide, it is the leading cause of cancer-related death in women in developing countries, and second only to lung cancer in more developed regions^1^.

Surgery, either breast-conserving surgery (BCS) or mastectomy, is the primary treatment for breast cancer. Despite its therapeutic intent, surgery causes physiological stress, which, along with anaesthesia^2^, can lead to transient immunosuppression during the perioperative period^3^. Such transient immunosuppression may lead to poorer immune detection of cancer cells^3^.

Postoperative bleeding requiring reoperation occurs in up to 4 per cent of women undergoing surgery for breast cancer^4^. Depending on the age of the patient and extent of primary surgery (mastectomy *versus* BCS)^5^, the use of certain prescription drugs (such as selective serotonin reuptake inhibitors (SSRIs) or glucocorticoids) increases the risk of postoperative bleeding requiring reoperation^5,6^. However, there is no evidence of an effect of SSRIs and glucocorticoid use on breast cancer recurrence^5–7^. Bleeding activates platelets, which can bind tumour cells, promoting immune evasion, angiogenesis, tumour cell survival and metastatic growth^8^. Cancer is associated with a hypercoagulable state^9,10^, with heightened platelet activation and a correlation with poor prognosis^11^. Thus, patients with breast cancer who develop postoperative bleeding requiring reoperation may be at increased risk of breast cancer recurrence.

This cohort study was conducted to investigate the association between bleeding occurring within 14 days of primary breast cancer surgery and the rate of recurrence among patients with breast cancer in Denmark.

## Methods

This study was approved by the Danish Data Protection Agency (Record 2007-58-0010), the Danish Medicines Agency and the Danish Breast Cancer Group (DBCG). The study is based on routinely collected registry data and according to Danish regulations does therefore not require separate ethical approval.

### Setting

This was a nationwide cohort study using Danish population-based registries. Denmark's national health service provides tax-supported healthcare to Danish citizens and permanent residents, including unrestricted access to hospital care and partial reimbursement for prescribed medications^12,13^. At birth or immigration, each person is assigned a unique civil personal registration number (CPR number) that allows unambiguous individual-level linkage among all Danish administrative and population-based registries, including medical registries^13^.

### Source population and data collection

The registry of the DBCG^14,15^ and the Danish National Patient Register (DNPR) was used to identify all women with an incident diagnosis of operable stage I–III breast cancer who underwent BCS or mastectomy between 1996 and 2008. To ensure correct retrieval of the exposure, defined as reoperation for postoperative bleeding within 14 days following primary breast cancer-directed surgery, patients were considered eligible for inclusion in the study if there was a difference of 1 day or less between the recorded date of primary surgery in the DNPR and DBCG database.

The DBCG has registered almost all women with invasive breast cancer in Denmark since 1977^16^. Data on tumour and patient characteristics are collected prospectively by treating physicians. The completeness of registration is approximately 95 per cent^16^. Patients registered in the DBCG database undergo regular follow-up examinations aimed at detecting recurrent disease^17^. The following information was obtained from the DBCG database: age and menopausal status at diagnosis, type of surgery, WHO histological tumour type and grade, lymph node status, tumour size, oestrogen receptor (ER) status, receipt of adjuvant chemotherapy, endocrine therapy (ET) and/or radiation therapy, and date and site of recurrence.

The DNPR has collected data on all non-psychiatric hospital admissions since 1977, and on all outpatient and emergency contacts since 1995. Data in the DNPR include the CPR number, one primary diagnosis, and one or more secondary diagnoses classified according to the ICD, as well as data on diagnostic and surgical procedures^18^.

The DNPR was used to retrieve information on reoperation for bleeding after surgery (*Table S1*, supporting information) within 14 days following primary surgery for breast cancer. Information was retrieved from the DNPR on potentially confounding other diseases (co-morbidity) registered up to 10 years before the breast cancer diagnosis. These were summarized using the Charlson Co-morbidity Index (CCI)^19^, modified to exclude breast cancer diagnoses. Co-morbidity prevalent on the date of breast cancer surgery was studied in order to detect diseases that could potentially confound or modify the association between bleeding after surgery and a later breast cancer recurrence^20–23^. These included: diabetes, liver disease, chronic pulmonary disease, peripheral and cerebral vascular disease, any other cancer, myocardial infarction and congestive heart failure (*Table S2*, supporting information). Information on death and emigration was retrieved from the Civil Registration System (CRS). The CRS, established in 1968, contains information on the vital status of all Danish citizens; it is updated daily^12^.

The National Prescription Registry has automatically recorded detailed information on all prescriptions redeemed at Danish community pharmacies since 1995^24^. Information is transferred electronically into the registry at the time of prescription redemption, so the validity of the registry is extremely high^25^. The registry contains detailed information on dispensed prescriptions, including full Anatomical Therapeutic Chemical codes, and date and quantity dispensed^24^. Data on drugs that potentially confound the association between bleeding and recurrence were retrieved, including simvastatin and aspirin, which may modify breast cancer prognosis^26,27^, and hormone replacement therapy (HRT) (*Table S3*, supporting information).

### Variables analysed

Age at diagnosis was categorized into decades. Histological grade was defined as low, moderate or high, based on WHO histological tumour type^28^. Stage was classified as I, II or III according to the UICC classification (6th edition)^29^. Lymph node status was defined according to number of involved nodes (0, 1–3, 4 or more). Tumour size was categorized as 20 mm or less, or over 20 mm. ER and adjuvant ET were summarized as: ER+/ET+, ER–/ET–, ER+/ET– or ER−/ET+. Surgery type was either mastectomy or BCS. Treatment with adjuvant chemotherapy was categorized dichotomously. Menopausal status at diagnosis was either premenopausal or postmenopausal, classified according to the DBCG.

Simvastatin and aspirin use were modelled as time-varying co-variables. Longitudinal prescription data were used to define time-updated exposure to these drugs. For each prescription, prescription duration was calculated as pack size (number of pills per pack) multiplied by the number of packages redeemed, assuming that a single pill was taken each day. In defining continuous use, a gap of 30 days was allowed from the end of one prescription (prescription start date + prescription duration) until the start of a new prescription. If a new prescription was redeemed within this window, then exposure was assumed to continue; if not, the patient was considered to have stopped the drug at the end of the 30-day grace period. The patient could later restart if there were further prescriptions. Finally, the resulting time-updated current medical exposure variable lagged by 1 year to allow the effect of the drug to accrue, as any effects on cancer are likely to be delayed, and to minimize confounding by indication. HRT was recorded as a baseline co-variable among women with at least 1 year of prescription history.

Breast cancer recurrence was defined according to the DBCG as any local, regional or distant recurrence, or cancer of the contralateral breast up to 10 years after the primary diagnosis^14^.

Follow-up began 14 days after primary breast cancer surgery (registered in the DNPR) and continued until breast cancer recurrence, death, emigration, 10 years of follow-up or 1 January 2013 (end of the study period), whichever came first.

### Statistical analysis

The proportion of patients with breast cancer who did or did not undergo reoperation for bleeding after surgery was calculated, by patient, tumour and treatment characteristics. Incidence rates (IRs) of recurrence per 1000 person-years were calculated, and the 5- and 10-year cumulative incidence of recurrence was estimated according to whether reoperation for bleeding after primary surgery had been undertaken. IRs were also categorized by time after surgery; recurrences developing within 2 years represented very early recurrence, those diagnosed at 2–5 years comprised early recurrence, and recurrences detected after 5 years represented late recurrence^30^.

The proportion of patients with breast cancer receiving mastectomy and BCS over time was calculated, as was the proportion needing a further operation over time.

Cox regression models with time from start of follow-up as the underlying time scale were used to compute crude and adjusted hazard ratios (HRs) for recurrence and associated 95 per cent confidence intervals for reoperation for postoperative bleeding. To model the cause-specific hazard, patients who died without a breast cancer recurrence were censored at the date of death. The adjusted model included the following potential confounders: age group at diagnosis, menopausal status, receipt of chemotherapy, lymph node status, tumour size, tumour grade, type of primary surgery, ER/ET status, co-morbidity, baseline HRT, and simvastatin and aspirin use after diagnosis (coded as time-varying co-variables lagging by 1 year). The analyses were stratified by age, receipt of chemotherapy, UICC stage and type of primary surgery. Crude and adjusted HRs according to site of recurrence were calculated.

The following sensitivity analyses were conducted: changing the 14-day window for reoperation and start of follow-up to 7 days after primary surgery; changing the inclusion criteria from no more than 1 day difference between the recorded date of primary surgery in the DNPR and the DBCG database to no more than 14 days and no more than 31 days; changing the study population to include only patients with stage I and II disease at diagnosis; and excluding patients with previous cancers.

Analyses were performed using Stata® version 13 (StataCorp, College Station, Texas, USA).

## Results

A total of 33 162 patients with breast cancer who underwent BCS or mastectomy between 1996 and 2008 were identified. The cohort consisted of 30 711 women after exclusion of 2425 women with more than 1 day difference in the date of surgery, or inconsistency in type of surgery, between the DNPR and the DBCG database, and 26 women who died or had an event registered before the start of follow-up (within 14 days after primary breast cancer surgery). The proportion of patients treated with BCS *versus* mastectomy increased in recent years accompanied by a decline in the rate of reoperation. Median follow-up was 7·0 years.

### Reoperation after surgery

Overall, 767 patients (2·5 per cent) had at least one reoperation within 14 days of the primary surgery. Compared with women who were not reoperated, a higher proportion of patients who underwent reoperation were postmenopausal (75·1 *versus* 72·5 per cent), and had co-morbid disease (CCI score of at least 1: 23·2 *versus* 20·1 per cent), a history of cerebrovascular disease (5·2 *versus* 3·4 per cent) and moderate-grade tumours (13·0 *versus* 11·0 per cent) (*Tables 1* and *2*). Reoperated patients were more likely to have undergone mastectomy than BCS as primary surgery (69·3 *versus* 57·9 per cent) and less likely to receive chemotherapy (28·7 *versus* 33·6 per cent). A higher proportion of patients without reoperation had stage III cancer (18·1 *versus* 14·0 per cent). Overall, 21·0 per cent of women in the breast cancer cohort had been prescribed aspirin or simvastatin during follow-up, and 41·6 per cent had been prescribed HRT before the breast cancer diagnosis. Reoperated patients were more likely to be concurrent aspirin users.

**Table 1:** Baseline characteristics of 30 711 patients diagnosed with stage I–III breast cancer in Denmark, 1996–2008, according to reoperation for postoperative bleeding

	All patients		Recurrence		Total person-years	
	Reoperation (*n***=** 767)	No reoperation (*n***=** 29 944)	Reoperation (*n***=** 126)	No reoperation (*n***=** 4643)	Reoperation	No reoperation
Overall					5241	200 685
Age at diagnosis (years)						
≤ 29	0 (0)	98 (0·3)	0 (0)	32 (0·7)	0	578
30–39	30 (3·9)	1357 (4·5)	8 (6·3)	311 (6·7)	217	9073
40–49	112 (14·6)	5070 (16·9)	20 (15·9)	838 (18·0)	850	36 701
50–59	237 (30·9)	8962 (29·9)	43 (34·1)	1455 (31·3)	1683	63 381
60–69	230 (30·0)	9258 (30·9)	31 (24·6)	1357 (29·2)	1550	61 232
70–79	131 (17·1)	4254 (14·2)	23 (18·3)	576 (12·4)	816	25 602
≥ 80	27 (3·5)	945 (3·2)	1 (0·8)	74 (1·6)	124	4118
Menopausal status at diagnosis						
Premenopausal	191 (24·9)	8226 (27·5)	36 (28·6)	1380 (29·7)	1411	59 317
Postmenopausal	576 (75·1)	21 704 (72·5)	90 (71·4)	3262 (70·3)	3830	141 296
Missing	0 (0)	14 (0·0)	0 (0)	1 (0·0)	0	72
Charlson Co-morbidity Index score						
0	589 (76·8)	23 913 (79·9)	110 (87·3)	3879 (83·5)	4152	165 150
1	107 (14·0)	3357 (11·2)	12 (9·5)	446 (9·6)	701	20 666
2	47 (6·1)	1683 (5·6)	2 (1·6)	209 (4·5)	265	9947
≥ 3	24 (3·1)	991 (3·3)	2 (1·6)	109 (2·3)	123	4922
Specific co-morbidities						
Myocardial infarction	15 (2·0)	356 (1·2)	1 (0·8)	42 (0·9)	79	1987
Congestive heart failure	18 (2·3)	385 (1·3)	1 (0·8)	35 (0·8)	74	1937
Vascular disease	21 (2·7)	518 (1·7)	1 (0·8)	68 (1·5)	127	2818
Cerebrovascular disease	40 (5·2)	1013 (3·4)	1 (0·8)	114 (2·5)	274	5597
Chronic pulmonary disease	39 (5·1)	1459 (4·9)	7 (5·6)	174 (3·7)	211	8467
Diabetes types 1 and 2	20 (2·6)	811 (2·7)	1 (0·8)	114 (2·5)	112	4491
Diabetes with organ damage	8 (1·0)	346 (1·2)	1 (0·8)	41 (0·9)	41	1824
Liver disease	10 (1·3)	250 (0·8)	1 (0·8)	33 (0·7)	29	1296
Any other cancer	24 (3·1)	1286 (4·3)	1 (0·8)	154 (3·3)	152	7360

Values in parentheses are percentages.

**Table 2:** Baseline tumour characteristics and treatments of 30 711 patients diagnosed with stage I–III breast cancer in Denmark, 1996–2008, according to reoperation for postoperative bleeding

	All patients		Recurrence		Total person-years	
	Reoperation (*n***=** 767)	No reoperation (*n***=** 29 944)	Reoperation (*n***=** 126)	No reoperation (*n***=** 4643)	Reoperation	No reoperation
Overall					5241	200 685
UICC stage						
I	284 (37·0)	10 852 (36·2)	36 (28·6)	1157 (24·9)	2095	78 669
II	367 (47·8)	13 465 (45·0)	52 (41·3)	1844 (39·7)	2539	92 554
III	107 (14·0)	5406 (18·1)	38 (30·2)	1620 (34·9)	550	28 262
Missing	9 (1·2)	221 (0·7)	0 (0)	22 (0·5)	57	1200
Tumour size (mm)						
≤ 20	438 (57·1)	17 190 (57·4)	57 (45·2)	2026 (43·6)	3160	121 891
> 20	321 (41·9)	12 544 (41·9)	67 (53·2)	2574 (55·4)	2021	77 267
Missing	8 (1·0)	210 (0·7)	2 (1·6)	43 (0·9)	60	1528
Lymph node status						
Negative	405 (52·8)	15 522 (51·8)	51 (40·5)	1807 (38·9)	2963	111 142
1–3 postive nodes	255 (33·2)	9147 (30·5)	38 (30·2)	1266 (27·3)	1731	62 306
≥ 4 positive nodes	104 (13·6)	5151 (17·2)	37 (29·4)	1563 (33·7)	533	26 735
Missing	3 (0·4)	124 (0·4)	0 (0)	7 (0·2)	14	502
Histological grade						
Low	621 (81·0)	24 522 (81·9)	105 (83·3)	3846 (82·8)	4218	163 024
Moderate	100 (13·0)	3301 (11·0)	11 (8·7)	548 (11·8)	714	22 769
High	44 (5·7)	1992 (6·7)	9 (7·1)	222 (4·8)	297	13 972
Missing	2 (0·3)	129 (0·4)	1 (0·8)	27 (0·6)	11	920
ER/adjuvant ET status						
ER–/ET–	134 (17·5)	5818 (19·4)	21 (16·7)	1174 (25·3)	892	35 750
ER+/ET–	184 (24·0)	7143 (23·9)	25 (19·8)	1087 (23·4)	1399	52 922
ER+/ET+	420 (54·8)	15 985 (53·4)	76 (60·3)	2177 (46·9)	2736	104 739
ER–/ET+	5 (0·7)	181 (0·6)	1 (0·8)	27 (0·6)	39	1330
Unknown	24 (3·1)	817 (2·7)	3 (2·4)	178 (3·8)	174	5944
Type of primary surgery						
Mastectomy	373 (48·6)	10 838 (36·2)	65 (51·6)	1867 (40·2)	2527	74 573
Mastectomy + RT	159 (20·7)	6486 (21·7)	34 (27·0)	1445 (31·1)	1074	41 563
BCS + RT	235 (30·6)	12 620 (42·1)	27 (21·4)	1331 (28·7)	1639	84 550
Adjuvant chemotherapy						
Yes	220 (28·7)	10 075 (33·6)	33 (26·2)	1628 (35·1)	1509	65 009
No	547 (71·3)	19 869 (66·4)	93 (73·8)	3015 (64·9)	3732	135 676
HRT before diagnosis						
Yes	316 (41·2)	12 452 (41·6)	37 (29·4)	1634 (35·2)	2220	83 790
No	451 (58·8)	17 492 (58·4)	89 (70·6)	3009 (64·8)	3021	116 896
Drugs taken during study period						
Simvastatin	148 (19·3)	6286 (21·0)	7 (5·6)	349 (7·5)	538	22 527
Aspirin (high and low doses)	190 (24·8)	6233 (20·8)	15 (11·9)	556 (12·0)	532	17, 613

Values in parentheses are percentages. ER, oestrogen receptor; ET, endocrine therapy; RT, radiotherapy; BCS, breast-conserving surgery; HRT, hormone replacement therapy.

### Recurrence after reoperation for bleeding

Overall, 4769 patients developed breast cancer recurrence during follow-up. The IR of recurrence was 24·0 (95 per cent c.i. 20·2 to 28·6) and 23·1 (22·5 to 23·8) per 1000 person-years for reoperated and non-reoperated patients respectively (*Table 3*). Regardless of reoperation status, the incidence rate was higher in the first 2 years after surgery, followed by a decrease (*Table S4*, supporting information). The 1-year IR of recurrence was 29·1 (13·1 to 44·1) and 21·3 (19·7 to 23·1) per 1000 person-years for reoperated and non-reoperated patients respectively. The IR of recurrence in the second year after primary surgery was 40·7 (28·3 to 58·6) per 1000 person-years for reoperated patients and 34·7 (32·6 to 36·9) per 1000 person-years for non-reoperated patients. After 5 years of follow-up, the IRs for patients who did and did not undergo reoperation were similar: 27·7 (22·6 to 33·9) and 26·9 (26·1 to 27·8) per 1000 person-years respectively. The 5-year cumulative incidence of recurrence was 12·8 and 12·5 per cent for patients with and without reoperation respectively; the 10-year cumulative incidence of recurrence was 19·9 per cent for reoperated patients and 18·9 per cent for non-reoperated patients (*Table S5*, supporting information).

**Table 3:** Incidence rates and hazard ratios for breast cancer recurrence, according to reoperation for postoperative bleeding, among 30 711 women diagnosed with stage I–III breast cancer in Denmark, 1996–2008 with follow-up to 31 December 2012

	No. of recurrences	Person-years	Crude incidence rate (per 100 000 person-years)	Unadjusted hazard ratio	Adjusted hazard ratio^*^
Overall (reoperation within 14 days)^†^					
No reoperation	4643	200 685	23·1 (22·5, 23·8)	1·00 (reference)	1·00 (reference)
Reoperation	126	5241	24·0 (20·2, 28·6)	1·05 (0·88, 1·25)	1·06 (0·89, 1·26)
Reoperation within 7 days^†^					
No reoperation	4650	201 520	23·1 (22·4, 23·7)	1·00 (reference)	1·00 (reference)
Reoperation	121	4995	24·2 (20·3, 28·9)	1·06 (0·88, 1·27)	1·08 (0·91, 1·30)

Values in parentheses are 95 per cent confidence intervals. Hazard ratios with 95 per cent confidence intervals are shown.

* Hazard ratios were adjusted for age (as a categorical variable), menopausal status at diagnosis (premenopausal, postmenopausal), lymph node status (negative, 1–3 positive nodes, at least 4 positive nodes), tumour size (20 mm or smaller, larger than 20 mm), histological grade (low, moderate, high), type of surgery, oestrogen receptor (ER) status and receipt of endocrine therapy (ET) (ER+/ET–, ER+/ET+, ER–/ET–, ER–/ET+), receipt of chemotherapy (yes, no), simvastatin use and aspirin use (both as time-varying co-variables lagging by 1 year), co-morbidity, and receipt of hormone replacement therapy before diagnosis (yes, no).

† The total number of patients with recurrence is not identical here because two patients died or developed a recurrence before the start of follow-up on day 14.

**Fig. 1:**
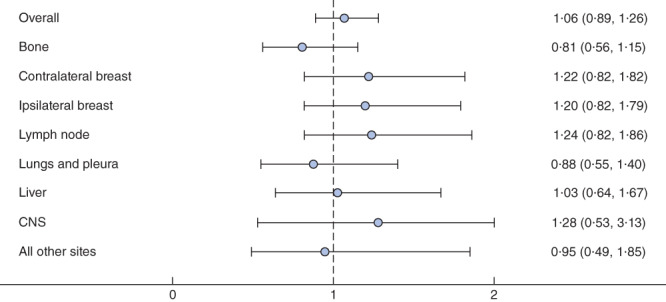
Forest plot showing associations between reoperation for postoperative bleeding and anatomical site of recurrence. Hazard ratios with 95 per cent confidence intervals are shown. Hazard ratios were adjusted for age (as a categorical variable), menopausal status at diagnosis (premenopausal, postmenopausal), lymph node status (negative, 1–3 positive nodes, at least 4 positive nodes), tumour size (20 mm or smaller, larger than 20 mm), histological grade (low, moderate, high), type of surgery, oestrogen receptor (ER) status and receipt of endocrine therapy (ET) (ER+/ET–, ER+/ET+, ER–/ET–, ER–/ET+), receipt of chemotherapy (yes, no), simvastatin use and aspirin use (both as time-varying co-variables lagging by 1 year), co-morbidity, and receipt of hormone replacement therapy before diagnosis (yes, no). CNS, central nervous system

**Fig. 2:**
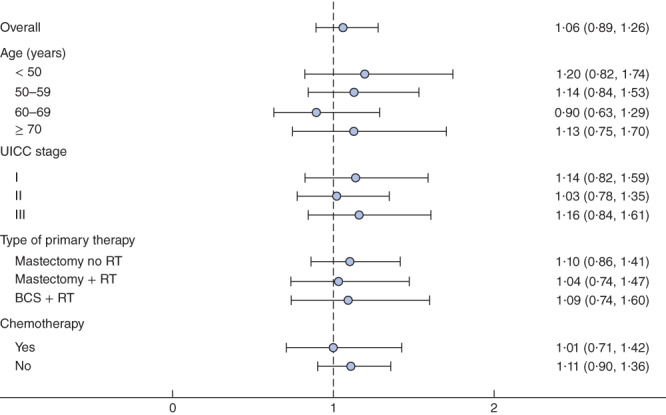
Forest plot showing associations between reoperation for postoperative bleeding and rate of breast cancer recurrence, stratified by age, UICC stage and type of primary therapy. Hazard ratios with 95 per cent confidence intervals are shown. Hazard ratios were adjusted for age (as a categorical variable), menopausal status at diagnosis (premenopausal, postmenopausal), lymph node status (negative, 1–3 positive nodes, at least 4 positive nodes), tumour size (20 mm or smaller, larger than 20 mm), histological grade (low, moderate, high), type of surgery, oestrogen receptor (ER) status and receipt of endocrine therapy (ET) (ER+/ET–, ER+/ET+, ER–/ET–, ER–/ET+), receipt of chemotherapy (yes, no), simvastatin use and aspirin use (both as time-varying co-variables lagging by 1 year), co-morbidity, and receipt of hormone replacement therapy before diagnosis (yes, no). RT, radiotherapy; BCS, breast-conserving surgery

Among 767 patients who underwent reoperation, there were 126 recurrences in 5241 person-years of follow-up. Among 29 944 women who did not undergo reoperation, there were 4643 recurrences in 200 685 person-years of follow-up. After adjusting for potential confounders, no association between bleeding after surgery and breast cancer recurrence was observed (adjusted HR 1·06, 95 per cent c.i. 0·89 to 1·26), regardless of time interval of exposure (7 or 14 days after primary operation) (*Table 3*). This lack of association did not change in sensitivity analyses in which the study population included only patients with stage I and II disease at diagnosis, patients with previous cancers were excluded, or patients with a difference in surgery date between the DNPR and DBCG database of no more than 14 days and no more than 31 days were included (*Table S6*, supporting information). The estimates did not vary by site of breast cancer recurrence (*Fig. 1*), and there was no evidence of effect modification in models stratified by age, tumour stage, type of primary surgery or receipt of chemotherapy (*Fig. 2*).

## Discussion

Previous research in Danish patients reported an association between re-excision (owing to insufficient surgical margins within 2 months of BCS) and increased risk of ipsilateral breast tumour recurrence^31^. This finding was, however, largely explained by residual disease^31^. The hypothesis for the present study was that patients who undergo reoperation for postoperative bleeding would be at increased risk of ipsilateral breast tumour recurrence. No evidence was found of an association between reoperation for bleeding after surgery and later breast cancer recurrence, regardless of time interval of exposure (7 or 14 days after the primary operation). Furthermore, the estimates did not vary in analyses stratified by clinical factors, the extent of primary surgery, or by site of breast cancer recurrence. A slight increase in early recurrence among reoperated patients was observed, but the estimates are imprecise.

Research suggests that mastectomy is associated with a higher risk of intraoperative bleeding and postoperative complications than BCS^32–34^. However, mastectomy alone and BCS combined with radiotherapy have equal efficacy in terms of preventing breast cancer recurrence^35^. Results from the present study show that the association of postoperative bleeding with breast cancer recurrence is not modified by the extent of primary surgery.

The associations observed for reoperation and breast cancer recurrence are not in line with those seen in patients undergoing surgery for gastrointestinal cancers. For example, intraoperative blood loss associated with surgery for upper gastrointestinal tract tumours decreases the activity of natural killer cells, which are the body's primary defence mechanism against cancer^36^. Research suggests that blood loss during surgery, regardless of whether blood transfusion is given, is a risk factor for peritoneal recurrence after curative resection of gastric cancer^37^. The mechanisms for the lack of concordance between these findings and those of the present study on breast cancer are unclear. Blood loss that can be controlled by further operation could be less extensive than blood loss that is sufficient to warrant a blood transfusion.

The main strengths of this study include its large size and population-based nationwide design within a setting of universal tax-supported healthcare. The prospective data collection reduced the potential for selection bias and ensured virtually complete follow-up. Furthermore, comprehensive data on potential confounders, including prescription drug data, were available. The crude estimates were quite similar to the adjusted estimates, and thus there was little evidence of confounding. It is also a strength that reoperation for bleeding after surgery has a surgical procedure code and is therefore well recorded in the database. Although the positive predictive value of this specific procedure code has not been assessed in the DNPR, it is expected to be high, as hospitals in Denmark are reimbursed only after registration of surgical procedures. It is nevertheless possible that other operative procedures could be misclassified as reoperation owing to postoperative bleeding. These include the codes for reoperation for postoperative infection or reoperation owing to other causes, which may include insufficient surgical margins (*Table S1*, supporting information). However, the latter misclassification is likely to bias the present findings away from the lack of effect of reoperation as residual disease is well known to be associated with recurrence^31^. The impact of postoperative infection on later breast cancer recurrence remains unclear.

Earlier studies^38,39^ used blood transfusion as a proxy for perioperative bleeding. However, in the case of breast cancer surgery, perioperative bleeding does not always result in blood transfusion. Furthermore, patients who receive blood transfusions are often sicker, with disseminated cancer, and more extensive co-morbidity.

The present study has some limitations. Information was missing on the extent of postoperative bleeding, in terms of actual blood loss. There was no information available on surgical complications that may have precipitated such bleeding. Another concern is the risk of selection bias due to exclusion of patients; however, the excluded patients were younger, had less advanced disease stages at diagnosis, and were less likely to receive mastectomy and ET (*Table S7*, supporting information). The sensitivity analyses also showed that the inclusion of these patients did not change the present findings (*Table S6*, supporting information). No information was available on the type of axillary surgery. However, from 2001 to 2006 the sentinel node technique was gradually introduced in Denmark^40^. During the study interval, all women with metastasis of any size in the axilla were offered axillary clearance level I + II as standard care.

Aspirin has been shown to decrease the risk of breast cancer mortality in some^26^, but not all^41,42^ studies, whereas simvastatin has been consistently associated with a decreased risk of breast cancer recurrence/mortality^43^. Information on prescribed aspirin was available, but it was not possible to account for aspirin bought over the counter. Aspirin formulations are available over the counter in Denmark but, if prescribed, almost exclusively done so in low doses for cardiovascular prevention. Over-the-counter aspirin is available only in small packs, and supplies for regular use are usually prescribed by physicians and reimbursable via the Danish National Health Insurance System. The proportions of total sales of low-dose aspirin dispensed by prescription, and thus captured in prescription registries, is high (92 per cent in 2012)^44^, so residual confounding regarding aspirin is expected to be a minor issue. No information on prescription compliance was available. In Denmark, patients pay part of the cost of redeemed prescriptions, so the estimates are likely to reflect actual use. Adjustment for prescribed aspirin and simvastatin did not change the findings. Finally, despite the large study size, reoperation for postoperative bleeding was relatively rare in this population and thus the precision of some of the estimates is low.

The findings of the present study have important clinical implications, and provide reassurance to patients and physicians that reoperation for postoperative bleeding does not increase the risk of breast cancer recurrence. Patients who undergo reoperation for bleeding are unlikely to need more aggressive adjuvant therapy. Breast cancer surgery involves a soft tissue surface and is often characterized by extensive dissection, which increases the risk of postoperative bleeding; the results may therefore be relevant to other soft tissue surgical procedures.

## Supplementary Material

bjs10592-sup-0001-Appendix **Table S1** ICD-10 codes for surgical procedures among women with stage I, II or III breast cancer in Denmark, 1996–2008
**Table S2** ICD codes for co-morbidities
**Table S3** Confounder drugs
**Table S4** Incidence of breast cancer recurrence for patients with stage I, II or III breast cancer in Denmark, 1996–2008, according to need for reoperation for postoperative bleeding, stratified by time after surgery
**Table S5** Five- and 10-year cumulative incidence of breast cancer recurrence for patients with stage I, II or III breast cancer in Denmark, 1996–2008, according to need for reoperation for postoperative bleeding
**Table S6** Breast cancer recurrences and hazard ratios for patients with stage I and II breast cancer, for patients without any previous cancers, and for patients with more than 1 day between the primary surgery date registered in the Danish National Patient Register and the Danish Breast Cancer Group database (in Denmark, 1996–2008), according to need for reoperation for postoperative bleeding
**Table S7** Comparison of baseline characteristics of patients retained in the cohort *versus* those excluded
