# Supplementary material for: Breast cancer recurrence after reoperation for surgical bleeding
Source: Br J Surg. 2017 Aug 7;104(12):1665–74. doi: 10.1002/bjs.10592 (PMC5655703; doi:10.1002/bjs.10592)
Supplement: bjs10592-sup-0001-Appendix — Table S1 ICD-10 codes for surgical procedures among women with stage I, II or III breast cancer in Denmark, 1996–2008 Table S2 ICD codes for co-morbidities Table S3 Confounder drugs Table S4 Incidence of breast cancer recurrence for patients with stage I, II or III breast cancer in Denmark, 1996–2008, according to need for reoperation for postoperative bleeding, stratified by time after surgery Table S5 Five- and 10-year cumulative incidence of breast cancer recurrence for patients with stage I, II or III breast cancer in Denmark, 1996–2008, according to need for reoperation for postoperative bleeding Table S6 Breast cancer recurrences and hazard ratios for patients with stage I and II breast cancer, for patients without any previous cancers, and for patients with more than 1 day between the primary surgery date registered in the Danish National Patient Register and the Danish Breast Cancer Group database (in Denmark, 1996–2008), according to need for reoperation for postoperative bleeding Table S7 Comparison of baseline characteristics of patients retained in the cohort versus those excluded [file bjs10592-sup-0001-appendix.docx]

**BJS10592**

**Reoperation for surgical bleeding in patients with breast cancer and breast cancer recurrence**

R. N. Pedersen, K. Bhaskaran, U. Heide-Jørgensen, M. Nørgaard, P. M. Christiansen, N. Kroman, H. T. Sørensen and D. P. Cronin-Fenton

**Table S1** ICD-10 codes for surgical procedures among women with stage I, II or III breast cancer in Denmark, 1996–2008

| **Postsurgical complications** | **ICD-10 codes** |
| --- | --- |
| Mastectomy  Breast-conserving surgery  Reoperation due to post-surgical bleeding | KHAC  KHAB  KHWD00, KHWE00 |
| Reoperation due to post-surgical infection | KHWB, KHWC |
| Reoperation due to other causes | KHWW |

**Table S2** ICD codes for co-morbidities

| **Charlson comorbidity category** | **ICD8** | **ICD10** | **Score** | **Comorbidity groups** |
| --- | --- | --- | --- | --- |
| Myocardial infarction | 410 | I21;I22;I23 | 1 | Myocardial infarction |
| Congestive heart failure | 427.09; 427.10; 427.11; 427.19; 428.99; 782.49 | I50; I11.0; I13.0; I13.2 | 1 | Congestive heart failure |
| Peripheral vascular disease | 440; 441; 442; 443; 444; 445 | I70; I71; I72; I73; I74; I77 | 1 | Vascular disease |
| Cerebrovascular disease | 430-438 | I60-I69; G45; G46 | 1 | Cerebrovascular disease |
| Dementia | 290.09-290.19; 293.09 | F00-F03; F05.1; G30 | 1 | - |
| Chronic pulmonary disease | 490-493; 515-518 | J40-J47; J60-J67; J68.4; J70.1;  J70.3; J84.1; J92.0; J96.1; J98.2; J98.3 | 1 | Chronic pulmonary disease |
| Connective tissue disease | 712; 716; 734; 446; 135.99 | M05; M06; M08; M09;M30;M31;  M32; M33; M34; M35; M36; D86 | 1 | - |
| Ulcer disease | 530.91; 530.98; 531-534 | K22.1; K25-K28 | 1 | - |
| Mild liver disease | 571; 573.01; 573.04 | B18; K70.0-K70.3; K70.9; K71; K73; K74; K76.0 | 1 | Liver disease |
| Diabetes type1    Diabetes type2 | 249.00; 249.06; 249.07; 249.09  250.00; 250.06; 250.07; 250.09 | E10.0, E10.1; E10.9  E11.0; E11.1; E11.9 | 1 | Diabetes types I & II |
| Hemiplegia | 344 | G81; G82 | 2 | - |
| Moderate to severe renal disease | 403; 404; 580-583; 584; 590.09; 593.19; 753.10-753.19; 792 | I12; I13; N00-N05; N07; N11; N14; N17-N19; Q61 | 2 | - |
| Diabetes with end organ damage type1  type2 | 249.01-249.05; 249.08  250.01-250.05; 250.08 | E10.2-E10.8  E11.2-E11.8 | 2 | Diabetes w/organ damage |
| Any tumor | 140-194 | C00-C75 | 2 | Any other cancer |
| Leukemia | 204-207 | C91-C95 | 2 | Any other cancer |
| Lymphoma | 200-203; 275.59 | C81-C85; C88; C90; C96 | 2 | Any other cancer |
| Moderate to severe liver disease | 070.00; 070.02; 070.04; 070.06; 070.08; 573.00; 456.00-456.09 | B15.0; B16.0; B16.2; B19.0; K70.4; K72; K76.6; I85 | 3 | Liver disease |
| Metastatic solid tumor | 195-198; 199 | C76-C80 | 6 | Any other cancer |
| AIDS | 079.83 | B21-B24 | 6 | - |

**Table S3** Confounder drugs

| **Potential confounder drugs** | **ATC codes** |
| --- | --- |
| Simvastatin | C10AA01 |
| Low-dose aspirin (75, 100 or 150 mg) | B01AC06 |
| High-dose aspirin, combinations | N02BA51 |
| Aspirin; 500 mg | N02BA01 |
| Hormone Replacement Therapy | G03C, L02AA, G03F, G03H, and G03D |

Prescription information on full Anatomical Therapeutic Chemical (ATC) codes were retrieved, and the date and quantity dispensed for relevant drugs.

**Table S4** Incidence of breast cancer recurrence for patients with stage I, II or III breast cancer in Denmark, 1996–2008, according to need for reoperation for postoperative bleeding, stratified by time after surgery

|  | **No reoperation** | | **Reoperation** | |
| --- | --- | --- | --- | --- |
| **Year** | **Recurrences (PY)** | **Incidence rate**†  **(95% CI)** | **Recurrences**  **(PY)** | **Incidence rate**†  **(95% CI)** |
| **0-1** | 630 (29.514) | 21.3 (19.7-23.1) | 22 (757) | 29.1 (13.1-44.1) |
| **1-2** | 974 (28,084) | 34.7 (32.6-36.9) | 29 (712) | 40.7 (28.3-58.6) |
| **2-5**  **0-5** | 1,943 (74,120)  3,547(131,718) | 26.2 (25.1-27.4)  26.9 (26.1-27.8) | 42 (1,892)  93 (3,361) | 22.2 (16.4-30.0)  27.7 (22.6-33.9) |
| **>5** | 1,096 (68,968) | 15.9 (15.0-16.9) | 33 (1880) | 17.6 (12.5-24.7) |

All estimates are unadjusted

† Per 1000 person-years

**Table S5** Five- and 10-year cumulative incidence of breast cancer recurrence for patients with stage I, II or III breast cancer in Denmark, 1996–2008, according to need for reoperation for postoperative bleeding

| **Patient characteristics** | **N**  **(total)** | **Recurrences** | | **5-year cumulative incidence (95% CI)** | **Recurrences** | **10-year cumulative**  **incidence (95% CI)** |
| --- | --- | --- | --- | --- | --- | --- |
| No Reoperation | 29,944 | 3,547 | 12.5 % (12.2 % - 12.9 %) | | 4,643 | 18.9 % (18.4 % - 19.5 %) |
| Reoperation | 767 | 93 | | 12.8 % (10.5 % -15.4 %) | 126 | 19.9 % (16.8 % - 23.4 %) |

All estimates are unadjusted

**Table S6** Breast cancer recurrences and hazard ratios for patients with stage I and II breast cancer, for patients without any previous cancers, and for patients with more than 1 day between the primary surgery date registered in the Danish National Patient Register and the Danish Breast Cancer Group database (in Denmark, 1996–2008), according to need for reoperation for postoperative bleeding

| **Exposure definition** | **Number of recurrences**  **(person-years)** | **Crude incidence rate (95% CI)*** | **Unadjusted**  **Hazard Ratio**  **(95% CI)** | **Adjusted**  **Hazard Ratio**  **(95% CI)**† |
| --- | --- | --- | --- | --- |
| **10-year Recurrences**  Stage I and II breast cancer patients only  No reoperation  Reoperation  Patients without previous cancers  No reoperation  Reoperation  Patients with ≤ 14 days difference in primary surgery date between DNPR and DBCG  No reoperation  Reoperation  Patients with ≤ 31 days difference in primary surgery date between DNPR and DBCG  No reoperation  Reoperation | 3,023 (172,423)  88 (4,691)  4,489 (193,325)  125 (5,089)  4,837 (208.732)  132 (5,509)  4,904 (213,096)  133 (5,579) | 17.5 (16.9-18.2)  18.8 (15.2-23.1)  23.2 (22.6-23.9)  24.6 (20.6-29.3)  23.2 (22.5-23.8)  24.0 (20.2-28.4)  23.0 (22.4-23.7)  23.8 (20.1-28.3) | 1.00  1.08 (0.87-1.33)  1.00  1.07 (0.89-1.27)  1.00  1.04 (0.88-1.24)  1.00  1.04 (0.88-1.24) | 1.00  1.05 (0.85-1.30)  1.00  1.09 (0.91-1.30)  1.00  1.06 (0.90-1.27)  1.00  1.07 (0.90-1.27) |

Abbreviations: CI, confidence interval; HR, hazard ratio

* Per 1000person-years

† HRs were adjusted for age (as a categorical variable), menopausal status at diagnosis (premenopausal or post-menopausal), lymph node status (negative, 1-3, ≥ 4), tumor size (**≤** 20, >20mm), histological grade (low, moderate, or high), surgery type, estrogen receptor (ER) status and endocrine therapy (ET) receipt (ER+/ET-, ER+ET+, ER-/ET-, ER-/ET+), receipt of chemotherapy (yes/no), simvastatin use and aspirin use (both as time-varying covariates lagged by 1 year), comorbidity, and receipt of prediagnostic hormone-replacement therapy (yes/no).

**Table S7** Comparison of baseline characteristics of patients retained in the cohort *versus* those excluded

simvastatin use and aspirin use (both as time-varying covariates lagged by 1 year), comorbidity, and receipt of prediagnostic hormone-replacement therapy (yes/no).

|  | **Final cohort** | | **Excluded patients** | |
| --- | --- | --- | --- | --- |
| Characteristic | N | % | N | % |
| **Age at diagnosis (years)** |  |  |  |  |
| ≤29  30-39  40-49  50-59  60-69  70-79  ≥80 | 98  1,387  5,182  9,199  9,488  4,385  972 | <1  4.5  17  30  31  14  3.2 | 14  143  592  807  637  229  29 | <1  5.8  24  33  26  9.4  1.2 |
| **Menopausal status at diagnosis** |  |  |  |  |
| Pre-menopausal  Post-menopausal  (Missing) | 8,417  22,280  14 | 27  73  <1 | 895  1,556  0 | 37  63  0 |
| **Charlson Comorbidity Index score** |  |  |  |  |
| 0  1  2  >=3 | 24,502  3,464  1,730  1,015 | 80  11  5.6  3.3 | 2,013  240  120  78 | 82  9.8  4.9  3.2 |
| **UICC stage** |  |  |  |  |
| I  II  III  (Missing) | 11,136  13,832  5,513  230 | 36  45  18  <1 | 1,245  913  266  27 | 51  37  11  1 |
| **Histologic grade** |  |  |  |  |
| Low  Moderate  High  (Missing) | 25,143  3,401  2,036  131 | 82  11  6.6  <1 | 1,910  249  274  18 | 78  10  11  <1 |
| **ER/adjuvant ET status** |  |  |  |  |
| ER-/ET-  ER+/ET-  ER+/ET+  ER-/ ET+  Unknown | 5,952  7,327  16,405  186  841 | 19  24  53  <1  2.7 | 489  881  957  11  113 | 20  36  39  <1  4.6 |
| **Type of primary surgery** |  |  |  |  |
| Mastectomy  Mastectomy + RT  BCS + RT | 11,211  6,645  12,855 | 36  22  42 | 646  338  1,467 | 26  14  60 |
| **Adjuvant chemotherapy received** |  |  |  |  |
| Yes  No | 10,295  20,416 | 34  66 | 1,643  808 | 33  67 |

Abbreviations: -, negative; +, positive; BCS, breast-conserving therapy; ER, estrogen receptor; ET, endocrine therapy; HRT, hormone-replacement therapy; RT, radiotherapy; UICC, Union for International Cancer control
